# Supplementary material for: Fast fitting of reflectivity data of growing thin films using neural networks
Source: J Appl Crystallogr. 2019 Nov 8;52(Pt 6):1342–7. doi: 10.1107/S1600576719013311 (PMC6878882; doi:10.1107/S1600576719013311)
Supplement: Supplementary file 1 [file j-52-01342-sup1.pdf]

JOURNAL OF APPLIED CRYSTALLOGRAPHY

Volume 52 (2019)

Supporting information for article:

Fast Fitting of Reflectivity Data of Growing Thin Films Using Neural Networks

Alessandro Greco, Vladimir Starostin, Christos Karapanagiotis, Alexander Hinderhofer, Alexander Gerlach, Linus Pithan, Sascha Liehr, Frank Schreiber and Stefan Kowarik

S1. Additional results of predictions on experimental data

Figure S1 to Figure S4 demonstrate the prediction performance of the neural network model on the datasets that were used to calculate the predictions accuracy values, but not shown in the main text. In general, the network performs worse on XRR curves that have less pronounced features, such as low thickness or high roughnesses. These curves are marked with shaded areas and were not included in the accuracy statistics.

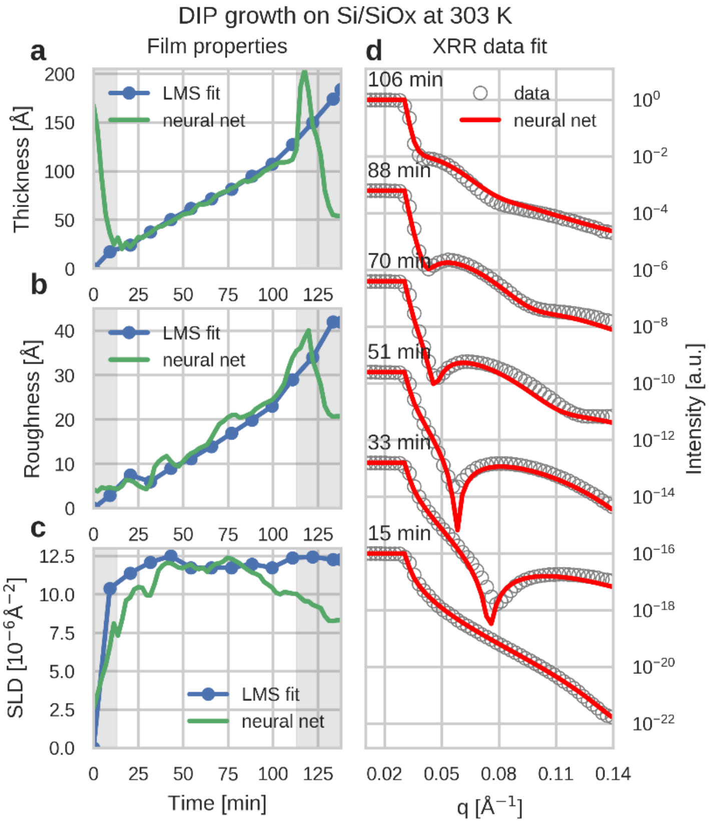

Figure S1 Fitting performance of the neural network model on a DIP film grown at 303 K with a deposition rate of 1.3 Å/min. (a-c) Comparison of the film parameters predicted by the neural network with results from least mean square fitting with human supervision at different times during growth. The shaded area marks films with low thickness (below 20 Å) or high roughness (above 30 Å) where data is difficult to fit for the NN. (d) Overlay of the experimental data with data simulated using the parameters predicted by the NN during different times during growth.

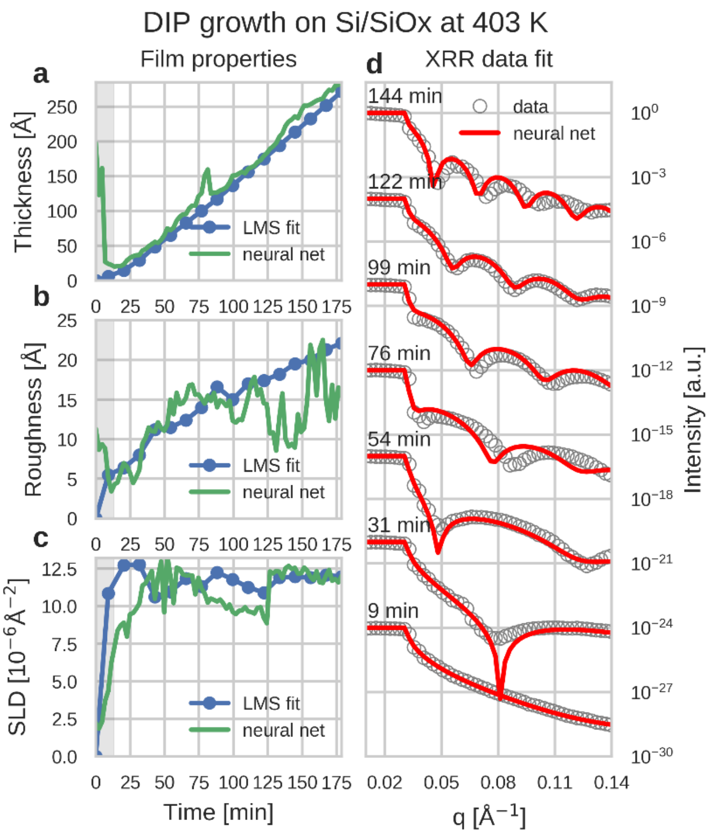

Figure S2 Fitting performance of the neural network model on a DIP film grown at 403 K with a deposition rate of 1 Å/min. (a-c) Comparison of the film parameters predicted by the neural network with results from least mean square fitting with human supervision at different times during growth. The shaded area marks films with low thickness (below 20 Å) where the data is difficult to fit for the NN. (d) Overlay of the experimental data with data simulated using the parameters predicted by the NN during different times during growth.

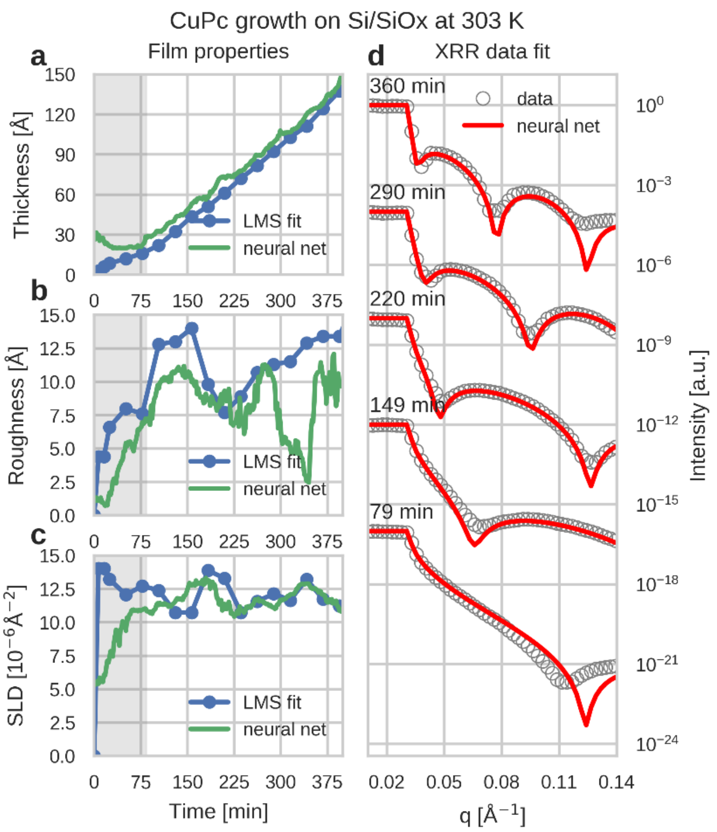

Figure S3 Fitting performance of the neural network model on a CuPc film grown at 303 K with a deposition rate of 0.4 Å/min. (a-c) Comparison of the film parameters predicted by the neural network with results from least mean square fitting with human supervision at different times during growth. The shaded area marks films with low thickness (below 20 Å) where the data is difficult to fit for the NN. (d) Overlay of the experimental data with data simulated using the parameters predicted by the NN during different times during growth.

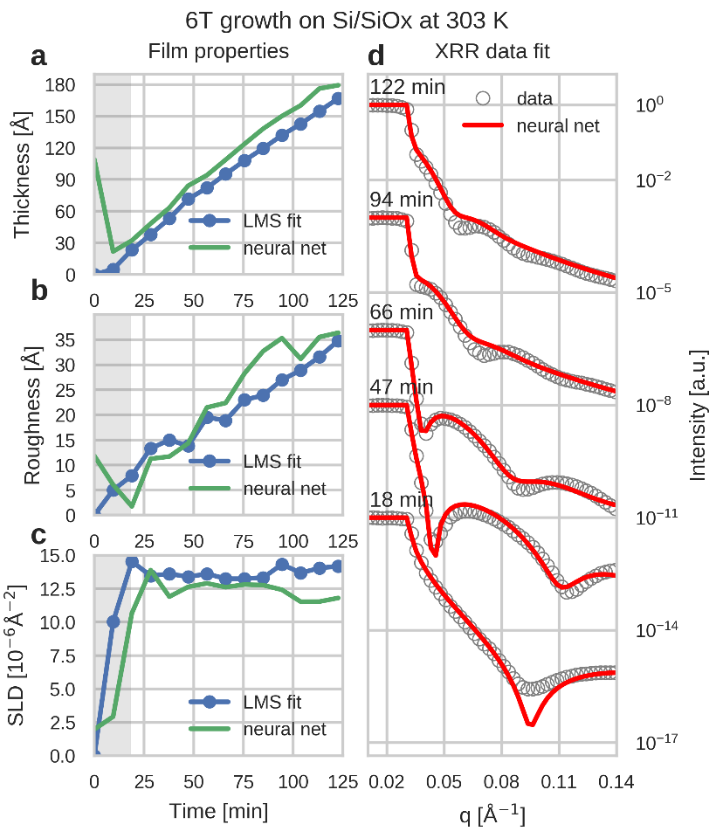

Figure S4 Fitting performance of the neural network model on a 6T film grown at 303 K with a deposition rate of 1.3 Å/min. (a-c) Comparison of the film parameters predicted by the neural network with results from least mean square fitting with human supervision at different times during growth. The shaded area marks films with low thickness (below 20 Å) where the data is difficult to fit for the NN. (d) Overlay of the experimental data with data simulated using the parameters predicted by the NN during different times during growth.

S2. Scattering length density profiles for the studied thin films at their final thickness

Figure S5 to Figure S9 show the SLD profiles of the five studied organic thin films at their final thickness, obtained via conventional (manual) least mean squares fitting.

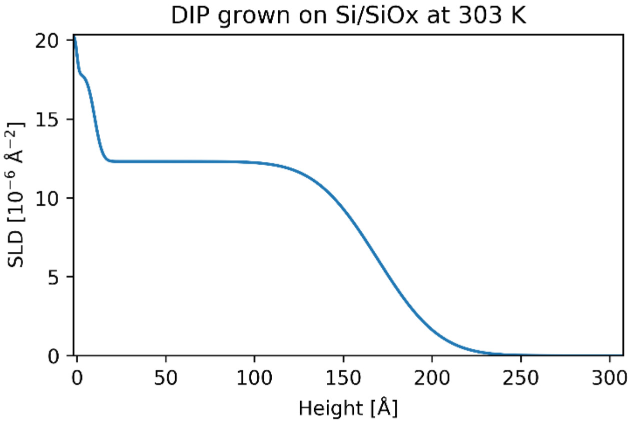

Figure S5 SLD profile of the DIP film grown on Si/SiO_x at 303 K with a deposition rate of 1 Å/min and a final thickness of about 180 Å.

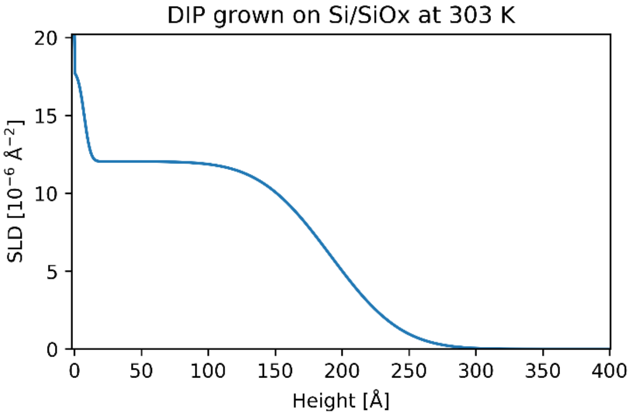

Figure S6 SLD profile of the DIP film grown on Si/SiO_x at 303 K with a deposition rate of 1.3 Å/min and a final thickness of about 170 Å.

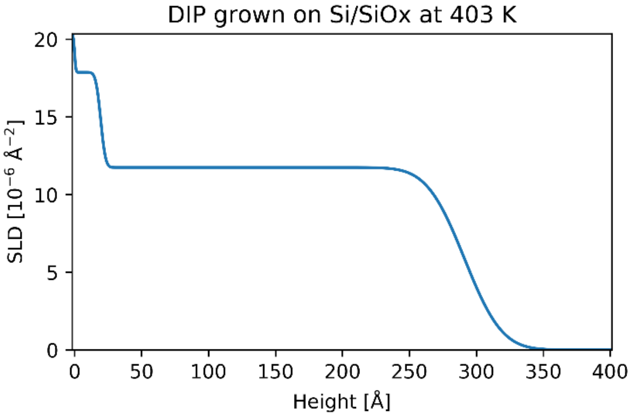

Figure S7 SLD profile of the DIP film grown on Si/SiO_x at 403 K with a deposition rate of 1 Å/min and a final thickness of about 270 Å.

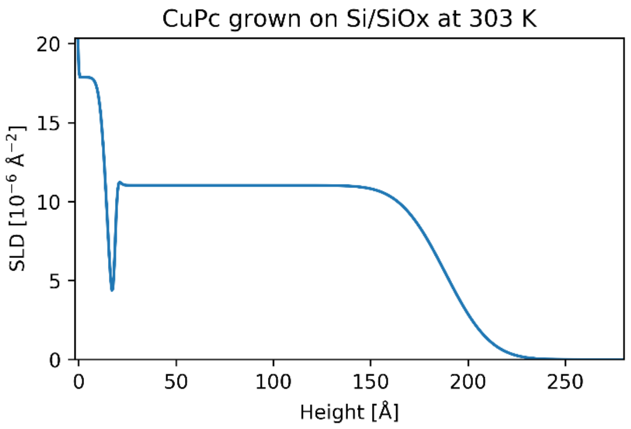

Figure S8 SLD profile of the CuPc film grown on Si/SiO_x at 303 K with a deposition rate of 0.4 Å/min and a final thickness of about 150 Å. A void layer was included between the substrate and the film to model the lower electron density at the substrate interface.

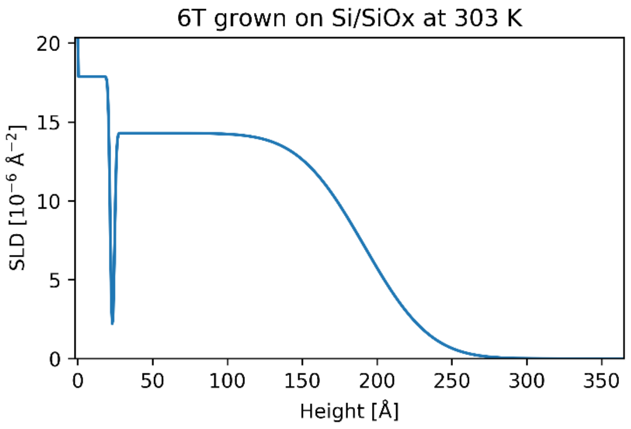

Figure S9 SLD profile of the 6T film grown on Si/SiO_x at 303 K with a deposition rate of 1.3 Å/min and a final thickness of about 165 Å. A void layer was included between the substrate and the film to model the lower electron density at the substrate interface.
